# Development of the Cybercrime Rapid Identification Tool for Adolescents

**DOI:** 10.3390/ijerph17134691

**Published:** 2020-06-30

**Authors:** Dennis Sing-wing Wong, Sai-fu Fung

**Affiliations:** Department of Social and Behavioural Sciences, City University of Hong Kong, Hong Kong, China; dennis.wong@cityu.edu.hk

**Keywords:** confirmatory factor analysis, cybercrime, adolescent, Chinese, student

## Abstract

Two studies were conducted to support the development of an eight-item Cybercrime Rapid Identification Tool (CRIT) and evaluate the psychometric properties of the proposed scale on samples of secondary school and university students. The CRIT was developed and evaluated in two cross-sectional studies with 2044 respondents from Hong Kong and China. Study 1 recruited 1533 secondary school students from Hong Kong with a mean age of 14.91 (SD = 1.77) years, and Study 2 recruited 511 university students from mainland China with a mean age of 20.41 (SD = 2.49) years. A stepwise confirmatory factor analytical approach was taken with further verification by exploratory factor analysis based on different samples. Factorial validity was further verified using confirmatory factor analysis. The analyses supported an eight-item scale with a two-factor structure. The eight-item CRIT was found to possess good internal consistency and concurrent validity. The studies offer promising support for the CRIT. It has the potential to advance epistemological methods and clinical research related to cybercrime prevention.

## 1. Introduction

According to the International Telecommunication Union [1], there are an estimated 4.1 billion internet users worldwide, equating to approximately 53.6% of the global population at the end of 2019. With the growing popularity of the internet, increasing attention has been paid to some of the negative issues surrounding internet usage [2,3,4,5]. In the United States, 95% of teenagers own a smartphone that allows for internet access, of which 59% have reported experience of cyberbullying in various forms, such as offensive name-calling and the spreading of false rumours [6,7]. In Hong Kong, according to a survey conducted by the Department of Health [8], over two-thirds of primary and secondary school students are spending up to three hours a day online. Studies have found that adolescents who spend a great deal of time on the internet are prone to having more negative online experiences [9,10]. Hence, this explorative study develops and evaluates a universal rapid identification tool to screen adolescents who may have a tendency to engage in deviant behaviour online, including the violation of internet rules and norms, illegal cyber-activities, cyberbullying, online harassment, hacking and trolling [11,12,13]. This tool will be particularly useful for frontline practitioners developing intervention programmes and for researchers designing epistemological surveys.

The rapid growth of cybercrime and deviant online subcultures can be mainly attributed to the accessibility and efficiency of the internet [14], with deviant online behaviour among adolescents having increased rapidly in the past decade in line with greater access to the internet [15,16,17]. The bogus identities and anonymity often permitted in virtual spaces further favour crime proliferation [14]. Compared to the consequences arising from performing traditional forms of crime, such as assault, those arising from deviant online behaviours are less apparent to young people [13]. 

With reference to the literature on social-cognitive effects [18], social learning theory [19,20] and self-control theory [21,22], a wide variety of risk factors contribute to cyber-deviance. Aggressive behaviour stimulus by socialisation can be predicted by an individual’s normative beliefs about aggression [23]. For instance, compared to the victim and uninvolved peer groups, bullies in traditional forms of bullying held significantly stronger normative beliefs about aggression [24]. Pornari and Wood [25] reported that students who participated in more severe or frequent aggressive behaviour had distorted thought patterns that grounded their aggressive behaviour through a tendency to justify or rationalise harmful acts to reduce self-censure [26,27]. Moral disengagement – legitimising an action by selectively applying moral censure—is more readily exercised by online perpetrators because there is a lower likelihood of consequences and any consequences are likely to be delayed; this is reinforced by the online feature of anonymity [9,28]. Baek [16] suggested that low self-control was a strong predictor of deviant online behaviour among adolescents. Other studies have also linked a lower level of self-control with participation in various forms of deviant and illegal online behaviour, including hacking [13,29,30], watching pornography [30], online harassment [13,31], and the unauthorised use of personal information [29,30,32].

The literature also suggests that engagement in deviant online behaviour is closely related to various measures of wellbeing and interpersonal relationships. For example, perceived self-esteem has an effect on online interactions [33]. A recent study also found that strengthening one’s resilience may deter the incidence of cybercrime [34]. In terms of interpersonal relationships, Zhang [35] explored the relationship between online culture, interpersonal relationships and cybercrime. Another study highlighted the adverse effect of social networking sites on forms of interpersonal intrusion, such as offline dating violence and cyberbullying [36]. Payne, et al. [37] also identified interpersonal support and digital skills as related to the risk of committing cyber-dependent crime. The above literature has provided insights into the potential threats presented by deviant online behaviour and the factors contributing to online deviance.

In short, we conceptualise cybercrime as the use of an internet access device as an instrument to further illegal ends. Measuring deviant online behaviour is a challenging task in at least two ways. First, the pace of technology is changing rapidly and it is difficult to find a measure that can fully explain netizens’ behaviour related to the latest information technology in the virtual world. Second, there are many existing internet addictive scales, which merely focus on measuring individual negative computing behaviours on a specific internet platform, such as an online game and social networking site [3]. To the best of our knowledge, however, there is no existing scale for identifying the risk of cybercrime among adolescents at an emergent stage. This paper is inspired by the criminology and social psychological literature discussed above with particular focus on the following two dimensions: 1) deviant online behaviour; 2) risk factors for engaging in cybercrime, namely general normative beliefs about aggression and moral disengagement. Hence, this paper reports on the development of a Cybercrime Rapid Identification Tool (CRIT) and the evaluation of its psychometric properties, including factorial validity, internal consistency and concurrent validity, using the latest psychometric evaluation tools and well-established construal-level scales.

To verify the validity of the CRIT, it was hypothesised that the proposed measure possesses a two-factor structure with good factorial validity (Hypothesis 1). The scale was expected to hold positive relationship with problematic internet usage and negative relationship satisfaction (Hypothesis 2). CRIT was also expected to demonstrate negative relationship with self-esteem, resilience and positive relationship satisfaction (Hypothesis 3).

## 2. Materials and Methods

### 2.1. Participants

Two cross-sectional studies were conducted, one in Hong Kong and one in mainland China, with a total of 2044 valid respondents. Study 1 was conducted from March to June 2018, with 1739 participants recruited. Among the participants were 1591 students from eight secondary schools in Hong Kong and 148 students recruited from the Hong Kong Federation of Youth Groups Crime Prevention Centre. There were two inclusive criteria for selecting the schools and respondents. First, all the respondents were full time school students. Second, we selected the schools according to the official government reported school banding, ranging from one to three. Band one indicates the top students in Hong Kong, while band three signifies the students with learning difficulties under the current steaming system. We recruited the students who were roughly equally distributed from band one, two and three schools. The gender distribution was 58.6% male and 41.4% female, with 99.1% of respondents in Form 1 to 6 (Form 1 = 19.0%, Form 2 = 21.3%, Form 3 = 20.6%, Form 4 = 18.1%, Form 5 = 19.0%, and Form 6 = 1.1%). After incomplete questionnaires were removed, Study 1 arrived at a valid sample of 1533, with a mean age of 14.91 (SD = 1.77) years. To ensure the proposed scale is not only applicable to the secondary school students in Hong Kong, Study 2 took place in April and May 2019. The research team recruited 511 undergraduate students with a mean age of 20.41 (SD = 2.49) years from a university in Guangzhou, China. The recruited sample comprised 85.5% female and 14.5% male respondents, which matched the demographic profile of the university’s student population. 

### 2.2. Procedure

In Study 1, anonymous paper-and-pencil questionnaires were distributed by the research team members, with the participants assured that their responses would be confidential. While completing the questionnaire, participants were not allowed to discuss it with others. They were also required to personally submit the questionnaire upon completion. In Study 2, students were invited to participate in this study on a voluntary basis through the university’s intranet system, with the questionnaire appended to a self-report smartphone-based application. The research process strictly adhered to international ethical standards, with written consent obtained from all the participants, including parental consent of the minors. The project was endorsed by the research ethics committee of the university.

The research team deployed a questionnaire based on 13 well-established measures related to time spent on the internet and online behaviour, attitude and habits, making up 97 items (in Study 1, *n* = 1533). The measures included time spent online [38], deviant online behaviours [15], cyber-safety awareness [39], cyber-security precautions [39], knowledge of illegal cyber-activities [39], cyber-victimisation [38], parent-child relationships [15], self-control [29], empathy [40], anger management [41], moral disengagement [28], proactive aggression [26] and general normative belief about aggression [27]. The questionnaires used in this study were translated into Chinese in adherence with the back-translation procedure and verified by the research team members, with particular attention paid to cross-cultural differences [42,43].

Several steps were taken in developing the CRIT and evaluating its factorial validity. To avoid the potential problem for over-fitting highlighted in the structural equation modelling (SEM) literature [44,45], the exploratory factor analysis (EFA) and confirmatory factor analysis (CFA) were computed based on different datasets obtained by randomly stratifying Study 1 (*n* = 1533) into two datasets (Sample 1, *n* = 767; Sample 2, *n* = 766). Each of the samples reflected the original sex ratio of the participants [46]. Sample 1 (*n* = 767) was then used to select the items for the CRIT using a stepwise confirmatory factor analytical (SCOFA) approach [47]. We used the SCOFA together with reference to the correlation between the items and by cross-checking the alpha if item was deleted value. We kept at least three items per factor to avoid the issue of model misidentification [48,49]. Then we further evaluated the proposed measure with the standard scale development procedure using EFA [50]. To evaluate the factor structure of the proposed scale, EFA was performed using principal component analysis with oblimin rotation [50,51,52]. The cut-off values adopted for the Kaiser-Mayer-Olkin (KMO) and Bartlett’s tests were >0.70 and *p* < 0.01, respectively. The identified factors possessed an eigenvalue greater than 1 and items had a factor loading of over 0.50 [48,53]. In addition, we also computed the McDonald’s omega hierarchical subscale with an observed value over 0.30 for each identified latent factor structure of the scale [54,55,56,57].

CFA was then performed to further evaluate the factorial validity of the eight-item CRIT based on Study 1 (Sample 2, *n* = 766) and Study 2 (Sample 3, *n* = 511) [58,59]. Diagonally weighted least squares (DWLS) was used as the estimation method due to the ordinal nature of the scale items [60,61,62]. The model fit and cut-off criteria were evaluated based on standard practices in SEM: model fit was considered good with a comparative fit index (CFI) and Tucker–Lewis index (TLI) of over 0.950, a standardised root mean square residual (SRMR) of under 0.08 and a root mean square error of approximation (RMSEA) of under 0.06 [58,63,64,65]. In addition to the above criteria, an acceptable model could also be indicated by χ^2/^df ≤ 3 [66,67].

Based on the data from Study 1 (*n* = 1533), the internal consistency of the eight-item CRIT was evaluated by Cronbach’s alpha and Cronbach’s alpha if-item-deleted [68], the corrected item-total correlation between the eight items [48,69], and the total McDonald’s omega [55,56,57].

Convergent validity was assessed based on the data from Study 2 (*n* = 511) with other construal-level scales reported. Based on the existing literature on cybercrime and cyberbullying, we expected the CRIT to be positively related to problematic internet usage [3,35,70] and negative relationship satisfaction [35,71,72]. Hence, the following well-established measures were used: the Problematic Internet Use Questionnaire (PIUQ) [73,74,75,76] and the Negative Semantic Dimension (NSD) from the Positive and Negative Semantic Dimensions of Relationship Satisfaction (PN-SMD) scale [77]. Again based on the literature, we expected the eight-item CRIT to be negatively related to self-esteem [33,78], resilience [79,80,81] and positive relationship satisfaction [35,71,72]. The following construal-level measures were used: the Rosenberg Self-esteem (RSE) scale [82,83,84,85], the Brief Resilience Scale (BRS) [86,87] and the Positive Semantic Dimension (PSD) from the PN-SMD scale [77]. The above analyses were conducted with IBM SPSS version 26.0 and the lavaan package version 0.6-5 [88] in R (3.6.3).

### 2.3. Measures

#### 2.3.1. Brief Resilience Scale

The BRS comprises six items using a 5-point Likert-type scale (from 1 = does not describe me at all to 5 = describes me very well) for the respondents to report how well each statement describes their behaviour and actions. Items 2, 4 and 6 are reversed in valence, such as ‘I have a hard time making it through stressful events.’ and ‘It is hard for me to snap back when something bad happens.’ Higher scores refer to high levels of resilience [87]. The Cronbach’s alpha of this measure was 0.71.

#### 2.3.2. Rosenberg Self-Esteem Scale

The RSE scale comprises 10 items to measure the participants’ self-esteem using a 4-point Likert-type scale (from 0 = strongly disagree to 3 = strongly agree). Five items (3, 5, 8, 9 and 10) were reverse-coded, e.g., ‘I feel I do not have much to be proud of.’, ‘I certainly feel useless at times.’ and ‘I wish I could have more respect for myself.’ Higher scores indicate a high level of self-esteem [82,83]. The Cronbach’s alpha for the RSE scale in this study was 0.76.

#### 2.3.3. Positive and Negative Semantic Dimensions of Relationship Satisfaction Scale

The PN-SMD comprises 14 items using an 8-point Likert-type scale (from 0 = not at all to 7 = completely) to measure two dimensions related to Positive Semantic Dimension (PSD) (items 1 to 7, α = 0.90), e.g., positive qualities like ‘enjoyable’ and ‘friendly’, and Negative Semantic Dimension (NSD) (items 8 to 14, α = 0.92), i.e., negative qualities like ‘discouraging’ and ‘miserable’. Higher scores indicate high levels of positive and negative relationship satisfaction, respectively [77].

#### 2.3.4. Problematic Internet Use Questionnaire

The PIUQ is evaluated on a 5-point Likert-type scale (from 1 = never to 5 = always) with 18 questions related to being obsessed with internet activities, (e.g., ‘How often do you daydream about the Internet?’), neglecting non-internet activities (e.g., ‘How often do you spend time online when you’d rather sleep?’), and being unable to stop using the internet (e.g., ‘How often do you try to conceal the amount of time spent online?’). High scores indicate a high level of problematic internet usage [73]. There were controversies related to the dimensionality of the scale. However, this was fully addressed in the recent studies [74,89,90]. Cronbach’s alpha for this measure was 0.86.

## 3. Results

### 3.1. Development of the Cybercrime Rapid Identification Tool and Its Factorial Validity

The items for constructing the CRIT were selected using SCOFA [47]. This was based on data collected from the online behaviour among adolescents questionnaire (Study 1), which comprised 97 items from well-established measures. Eight items with a two latent factor structure were identified based on the conceptual themes related to deviant online behaviour (Items 1 to 4) [15], general normative belief about aggression (Item 5) [27], moral disengagement (Item 6) [28], self-control (Item 7) [41] and proactive aggression (Item 8) [26]. The identified items were further verified based on the EFA results. Factor analysis showed a KMO value of 0.86 (χ^2^ =2458.88, *p* < 0.001) for the 8-item CRIT from the data from Study 1 (Sample 1, *n* = 767). Table 1 shows the results of EFA using principal component analysis with oblimin rotation extracting two factors. The CRIT accounted for 66.36% of the total variance. The explanatory power of the factors in relation to the total variance was as follows. The first factor, related to deviant online behaviour (DOB), comprised four items with factor loadings ranging from 0.71 to 0.83 and explanatory power of 18.84% (eigenvalue = 1.51). The second factor, related to morality, control and aggression (MCA), comprised four items with factor loadings ranging from 0.80 to 0.87 and explained 47.52% of the variance (eigenvalue = 3.80). The McDonald’s omega results replicated the EFA findings: ω_hs.DOB_ and ω_hs.MCA_ showed relatively large omega hierarchical subscale values of 0.44 and 0.46, respectively [54]. Overall, a two latent factor structure was indicated.

**Figure 1:**
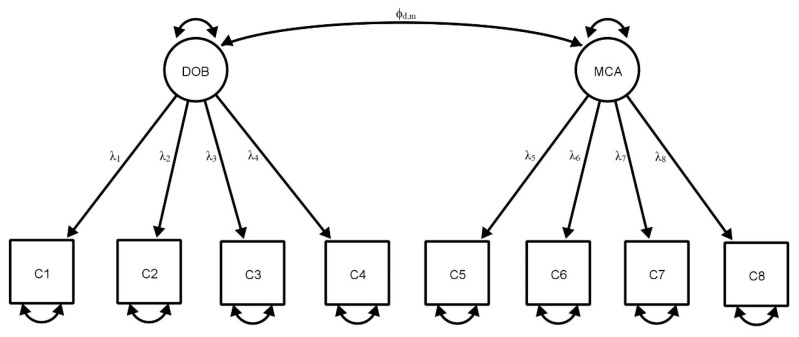
Final standardised model of the 8-item Cybercrime Rapid Identification Tool; DOB = deviant online behaviour; MCA = moral, control and aggression.

The CFA results for the 8-item CRIT using data from Sample 2 (*n* = 766) and Sample 3 (*n* = 511) are presented in Table 2 (see Figure 1 for the estimated model). The CFA results indicated that all models fulfilled the criteria for good model fit. As such, the CFA results in Sample 2 (*n* = 766) replicated the factor structure suggested by the above EFA results derived from Sample 1 (*n* = 767), χ^2^ (30.22)/19 = 1.59, SRMR = 0.03, CFI = 0.99, TLI = 0.99 and RMSEA = 0.03. The CFA results for the whole sample in Study 1 (*n* = 1533) yielded similar outcomes. The results from Study 2 (Sample 3, *n* = 511) also indicated good model fit, χ^2^ (21.23)/19 = 1.12, SRMR = 0.03, CFI = 0.99, TLI = 0.99 and RMSEA = 0.02. In short, the results indicated that the eight-item CRIT with a two-factor structure possessed good factorial validity.

### 3.2. Internal Consistency.

Table 3 shows the means, standard deviations, skewness, kurtosis, correlations, corrected item-total correlations and Cronbach’s alpha if-item-deleted for the proposed eight items of the CRIT from the data obtained from Study 1 (*n* = 1533). The mean score of CRIT was 15.16 (SD = 5.21). The corrected item-to-total correlations in the eight-item CRIT ranged from 0.47 to 0.66, suggesting appropriateness for constructing a scale. The Cronbach’s alpha and McDonald’s omega total of the scale were 0.85 and 0.88, respectively, indicating a measure with high internal consistency.

### 3.3. Concurrent Validity

Table 4 shows the correlation coefficients between CRIT and the selected well-established scales for testing concurrent validity, using the data from Study 2 (*n* = 511). The results supported the expectation that CRIT would display significant weak-to-moderate positive relationships with the Problematic Internet Use Questionnaire (PIQU) (*r* = 0.27, *p* < 0.001) and NSD (*r* = 0.39, *p* < 0.001), and significant weak-to-moderate negative relationships with PSD (*r* = −0.29, *p* < 0.001), RSE (*r* = −0.15, *p* < 0.001) and BRS (*r* = −0.18, *p* < 0.001). The results therefore indicate good concurrent validity for the 8-item CRIT.

## 4. Discussion

This study pioneers the development of a rapid identification tool that may connect to actual cybercrime at an emergent stage. The proposed eight-item CRIT was found to possess good psychometric properties which supporting the hypothesis 1. The scale returned alpha coefficient and omega total value above the acceptable range in both Study 1 (*α* = 0.85; ω = 0.88) and Study 2 (*α* = 0.88; ω = 0.90) [50,55,56,57]. The EFA and CFA results supported a measure with a two latent factor structure, with the factors being involvement in deviant online behaviour (DOB) and personal traits related to morality, control and aggression (MCA). All models in the CFA suggested good model fit for the CRIT with a two factor structure, fulfilling the standard criteria used in the SEM literature [58,64,67]. The scale also demonstrated good concurrent validity, with significant positive relationships with problematic internet usage and negative relationship satisfaction and significant negative relationships with resilience, self-esteem and positive relationship satisfaction. Thus, hypotheses 2 and 3 are accepted. Those findings are consistent with the normative arguments and epistemological findings highlighted in the existing literature related to internet-based deviant behaviours, such as cyber-loafing [78], cyberbullying [72,79], racial typification [70], and cyber-ostracism [81].

The scale can provide a handy identification tool not only for Chinese populations, but also for people in different cultures and societies. The scale was developed mainly from the Study 1 (*n* = 1533) sample of secondary school students in Hong Kong [91]. Due to the unique historical context, Hong Kong students are mostly bilingual with proficiency in Chinese and English. This distinctive cultural pattern of ‘East meets West’ and the high rate of internet use makes Hong Kong an ideal research setting for studying online behaviour [92,93,94]. In addition, this pioneering study also used two versions of the CRIT, one written in traditional Chinese (Study 1) and one written in simplified Chinese (Study 2). The simplified Chinese characters are commonly used in mainland China, whereas traditional Chinese is the written language used in Hong Kong, Taiwan and among other Chinese diasporas. This research design are commonly used in many cyber-behaviour studies using the same language in different societies [95,96]. Hence, this study provides evidence supporting the use of the CRIT in different societies.

This study also contributes to the application of the CRIT in the following ways. The identified two latent factor structure of the scale, comprising involvement in deviant online behaviour (DOB) and traits related to morality, control and aggression (MCA), can be adjusted through socialisation by different agencies operating specific intervention programmes in school [26] using a reactive-proactive model [97].

The study also takes a step forward in validating two less commonly recognised risk factors for engaging in cybercrime and cyber-deviance, namely general normative beliefs about aggression and moral disengagement. Early intervention targeting these factors could be made by institutions and caregivers (e.g., schools, parents and social workers) to reduce the risk of adolescents engaging in cybercrime and victimisation [98,99,100].

The findings should be considered alongside the following three potential limitations. First, the use of self-report questionnaires could have resulted in underreporting of deviant behaviour and misunderstandings of some items. Second, the current study does not contain enough empirical data to recommend any cut-off values in this proposed tool. Also, samples from Study 1 and 2 differ significantly in age. Further study is required to obtain normative data with a large and representative sample from the wider population. Third, there is currently lack of intervention programmes, external validation criteria, and clinical data to evaluate the effectiveness and sensitivity of the rapid identification tool. Although the eight items are certainly of paramount significance to identifying at-risk adolescents, the practical effectiveness of the tool requires examination.

## 5. Conclusions

This initial study strived to provide early data on a detailed way of developing a measure to identify adolescents at risk for cybercrime. For a more in-depth analysis of the risks, future research may consider conducting mediation or moderation analyses to assess the validity of the rapid identification tool on samples differing from those used here on various characteristics, such as age and gender. To validate the self-reported findings, other forms of deviant online behaviour assessment, such as behavioural observations of adolescents by parents and teachers, could be simultaneously carried out in future research. A longitudinal study could also be adopted to measure the effectiveness of the rapid identification tool. 

## Figures and Tables

**Table 1:** Factor loading resulted from exploratory factor analysis.

Item	DOB	MCA
1. I have downloaded illegal software.	**0.77**	−0.05
2. I have spread false information in an Internet board.	**0.82**	0.01
3. I have hacked other people’s computers or websites.	**0.83**	0.01
4. I have used other people’s Internet ID or resident registration number without permission.	**0.71**	0.06
5. In general, it is OK to take your anger out on others by using physical force.	0.03	**0.85**
6. Teasing someone does not really hurt them.	−0.01	**0.84**
7. If things I do upset people, it’s their problem, not mine.	−0.07	**0.87**
8. I threaten others because then it’s me who decide.	0.07	**0.80**

DOB = deviant online behaviour; MCA = moral, control and aggression.

**Table 2:** Factor loadings and fit indices in confirmatory factor analysis (CFA) for the Cybercrime Rapid Identification Tool (CRIT), by study (see Figure 1).

Factor/Question Number		Study 1	Study 1	Study 2
		(Sample 2)	(Combo)	Sample 3
DOB				
1	λ_1_	0.70	0.71	0.56
2	λ_2_	0.81	0.85	0.89
3	λ_3_	0.90	0.89	0.96
4	λ_4_	0.77	0.76	0.86
MCA				
5	λ_5_	0.85	0.86	0.67
6	λ_6_	0.77	0.79	0.94
7	λ_7_	0.76	0.78	0.80
8	λ_8_	0.81	0.84	0.85
Latent factor covariance				
DOB ~ MCA	φ_d,m_	0.61	0.61	0.87
Model fit				
*n*		766	1533	511
RMSEA		0.03	0.02	0.02
RMSEA 90% CI		0.02–0.05	0.01–0.03	0.00–0.04
SRMR		0.03	0.02	0.03
χ^2^ (df = 19)		30.22	36.24	21.23
χ^2^/df		1.59	1.91	1.12
CFI		0.99	0.99	0.99
TLI		0.99	0.99	0.99

Combo = sample 1 plus sample 2 from Study 1 (*n* = 1533); DOB = deviant online behaviour; MCA = moral, control and aggression.

**Table 3:** Descriptive statistics and items correlations for the CRIT items.

Item	1	2	3	4	5	6	7	8
1	–	0.47	0.48	0.45	0.28	0.23	0.22	0.27
2	0.54	–	0.64	0.50	0.35	0.30	0.27	0.36
3	0.53	0.64	–	0.50	0.39	0.29	0.27	0.36
4	0.48	0.53	0.53	–	0.34	0.30	0.27	0.34
5	0.31	0.35	0.36	0.36	–	0.61	0.59	0.64
6	0.27	0.32	0.30	0.34	0.63	–	0.58	0.56
7	0.24	0.29	0.28	0.30	0.59	0.58	–	0.58
8	0.30	0.37	0.37	0.37	0.65	0.58	0.59	–
								
Mean	1.84	1.58	1.44	1.72	2.09	2.20	2.27	2.03
SD	1.10	0.85	0.81	0.94	0.95	0.99	0.95	0.94
Skewness	1.17	1.44	1.90	1.23	0.52	0.45	0.31	0.50
Kurtosis	0.46	1.78	3.39	0.99	−0.20	−0.23	−0.20	−0.35
r*_it_*	0.47	0.58	0.59	0.54	0.66	0.59	0.57	0.64
*a_iid_*	0.87	0.86	0.86	0.86	0.85	0.85	0.85	0.85

All correlations are significant at the 0.001 level (2-tailed); Lower triangle for Spearman correlations; upper triangle for Pearson correlations; r*_it_* = Corrected item-total correlations; *a_iid_* = Cronbach’s alpha, if item deleted.

**Table 4:** Correlations between CRIT and its latent factor structure in relation to other construct-related scales.

Scale	CRIT	CRIT: DOB	CRIT: MCA
Problematic Internet Use Questionnaire	0.27	0.23	0.26
Negative Semantic Dimension	0.39	0.34	0.37
Positive Semantic Dimension	−0.29	−0.24	−0.29
Rosenberg Self-esteem	−0.15	−0.13	−0.15
Brief Resilience Scale	−0.18	−0.17	−0.16

All correlations are significant at the 0.001 level (2-tailed); PIUQ = Problematic Internet Use Questionnaire; NSD = Negative Semantic Dimension; PSD = Positive Semantic Dimension; RSE = Rosenberg Self-esteem; BRS = Brief Resilience Scale; DOB = deviant online behaviour; MCA = moral, control and aggression.
